# Practical Treatise on Hernia, Displacement, and Diseases of the Womb, &c.

**Published:** 1842-07

**Authors:** 


### ART. VIII.

1. *Traité Pratique des Hernies, Déplacements et Maladies de la Matrice, &c.* Par P. L. VERDIER, Chirurgien Herniaire de la Marine Royale, des Hôpitaux Militaires de France, &c.—*Paris*, 1840. 8vo, pp. 740.

*Practical Treatise on Hernia, Displacement, and Diseases of the Womb, &c.* By P. L. VERDIER.—*Paris*, 1840.

2. *Leçons Cliniques sur les Hernies, faites à l'Amphithéâtre du Bureau Central des Hôpitaux Civils de Paris, en* 1839-40. Par J. F. MALGAIGNE, Professeur agrégé de la Faculté de Médecine de Paris; et recuéllies sous ses yeux par M. ED. GELEZ, Interne des Hôpitaux de Paris.—*Paris*, 1841. 8vo, pp. 238.

*Clinical Lectures on Hernia, delivered in* 1839-40. By J. F. MALGAIGNE; collected under the Professor's superintendence by M. E. GELEZ. —*Paris*, 1841.

3. *A Treatise on Ruptures.* By W. LAWRENCE, F.R.S. Surgeon Extraordinary to the Queen; Surgeon to St. Bartholomew's Hospital, &c. Fifth Edition.—*London*, 1838. 8vo, pp. 632.

4. *Traité de Pathologie Externe, et de Médecine Opératoire.* Par AUG. VIDAL (DE CASSIS), Chirurgien de l'Hôpital de Lourcine, &c. Tome Cinquième.—*Maladies de l'Abdomen.*—*Paris*, 1841.

*Treatise on Surgery.* By AUG. VIDAL. Vol. V.—*containing the Section on Diseases of the Abdomen.*—*Paris*, 1841.

THE discrepancy which exists in regard to the general statistics of hernia, as supplied from various sources and by different authorities, is, on first inspection, not a little puzzling: but reflection will trace it to its proper source—viz., the generalizing from observations which are either too limited, or of too comprehensive a character. Sufficient importance has not been attached to circumstances of age, occupation, social position, climate, &c.; and the consequence is the contradictory statements to which we allude. Thus we find, as quoted by both Lawrence and Malgaigne, that the returns of the proportion of the ruptured, to the whole population, varies very greatly, as given by Arnaud, Turnbull, Bordenave, Juville, &c. We cite these after our French author, previously to accompanying him and his contemporary, Verdier, into the more satisfactory details with which they supply us.

The proportions of Arnaud and Bordenave present us with extremes; the former affirming that one in eight of the whole population are affected with hernia, whilst the latter states that one in a hundred is the average. The results obtained by Mr. Turnbull, surgeon to the London Truss Society, after "the most diligent and general enquiries throughout the kingdom," are, that the proportion of ruptured persons is one in fifteen. We need scarcely observe that *enquiries*, such as those alluded to, must necessarily be limited at the least as to station of life, as well as sex; unless, indeed, the information obtained second-hand from practitioners and truss-makers—sources too indirect and equivocal for trustworthy statistics. M. Louis's computation was made from the Parisian hospitals, in which the circumstances of sex, age, and employment were, to a certain extent, indicated; but of course the inmates of hospitals do not afford us the proper means of generalizing respecting a whole population: his numbers are, in the Salpêtrière (women), three per cent.; in the Bicêtre (men), six per cent.; in the Invalides (old soldiers), seven per cent.; in la Pitié (children and youths), two per cent. To these conclusions M. Malgaigne raises objections, asserting rather amusingly that he does not believe M. Louis could have had access to the persons of all the old women of the Salpêtrière, or the veterans of the Invalides. We feel as readily disposed to admit the testimony of Juville, a Parisian truss-maker, as any that we have already cited; his range of observation has at any rate been sufficiently extensive, and is probably not obnoxious to the objection that it excluded any particular class or either sex. In the north of Europe and Germany he computes that one in thirty are ruptured; in Italy and Spain one fifteenth of the population; and one twentieth in France and England. Dr. Knox, in his observations on this subject, in the Edin. Med. and Surg. Journal, states that he examined eighty-six persons of the lower orders without finding one case of hernia. So much for these unsatisfactory results: now let us turn to where we at the least have promises of something more definite.

In reference to sex, we extract M. Malgaigne's introductory remarks and table.

"We have sought to avoid all sources of objection, to discard all such equivocal means of research, and, in short, to have periods of comparison and proper control over our resources, by forming three series of observation corresponding to three several epochs; thus, in October and November 1835, we had collected 435 observations, of which twenty-five were cases of prolapsus. Deducting these cases, 410 remained which were herniæ; of these 335 were in men, and 75 in women.

| | | | | | |
|---|---|---|---|---|---|
| In 1836 of | 2767 | herniæ, | 2203 were in men, | 564 | in women. |
| 1837 „ | 2373 | „ | 1884* „ | 489 | „ |

The mean result is about four to one."

The tables of M. Verdier are copious, and include every point connected with the statistics of hernia; and the number of cases upon which his classifications are founded is satisfactory, viz., 1226. We have no reason or right to doubt that they are authentic, and shall therefore avail ourselves of them as such. We find, in the summing up of his

* In the original, 2884; evidently a misprint.—ED.

third table (p. 228), that the gross number of each sex afflicted with rupture is, of males 976, and females 250; a proportion which agrees accurately with that of M. Malgaigne. We shall have occasion, in our discussion of the various forms of hernia, to refer to the details of this and others of M. Verdier's statistical reports. Lastly, we turn to Mr. Lawrence, who gives us an abstract from the register of patients relieved by the London Truss Society, during twenty-eight years. Of 83,584 patients, 67,798 were males, and 15,786 were females. (p. 11.) In this it will be observed that the proportion of females falls very little short of that afforded by the results of our French authors: we may therefore assume that the relation of *four to one* is a correct average as regards the two sexes.

The age at which hernia is most frequent next occupies the attention of M. Malgaigne. After observing that the London Society, already referred to, possesses the only documents that he is acquainted with, where an account of ages has been kept, he gives way to a burst of lamentation that "people but little advanced in civilization should take so little care of preserving a knowledge of their ages;" and thence proceeding directly to generalize upon an amusing story told by one of his own countrymen respecting an English "savant," who, in making some researches in which age was a desideratum, was unable to ascertain that of either his wife or servant,—concludes that probably England may justly be included in the "barbarous" category, and that the London Society's tables are not therefore deserving of the confidence they would seem to claim. We do not pretend to decide upon the relative chronological faculties and facilities of the two nations, but surely our author gives us a bad sample of philosophical deduction, while he is, at the same time, calling upon us largely to give credence to the results of his own impartial investigations. For all practical ends the *exact* age of individuals is by no means essential for statistical purposes in a question like the present: we therefore think the following table of the Truss Society both interesting and important. Of the eighty-three thousand cases already alluded to, 5448 had congenital hernia; 7299 were relieved with trusses under ten years of age; 4551 between ten and twenty; 8715 between twenty and thirty; 13,614 between thirty and forty; 15,627 between forty and fifty; 14,169 between fifty and sixty; 9761 between sixty and seventy; 3866 between seventy and eighty; 442 between eighty and ninety; 23 between ninety and a hundred. (Lawrence, p. 13.) As we have not space to give M. Verdier's table in detail, we insert the account of his 1226 cases as they occurred between the ages set forth in the foregoing table. Under ten years of age the number was 973; from ten to twenty 152; from twenty to thirty 57; from thirty to forty 25; from forty to fifty 11; from fifty to seventy 8. (Verdier, p. 237.) The discrepancy between these accounts proves how difficult it is to arrive at satisfactory results where the object of investigation cannot be reduced to one of simple observation, unembarrassed by circumstances such as neglect, ignorance of the existence of disease, &c., on the part of patients.

But let us turn to the more elaborate enquiry of M. Malgaigne; he does not favour us with a tabular view, but informs us that "his cases are distributed in three series, and that these three series coinciding, his conclusions are thereby greatly strengthened."

"If, from the total number of herniæ which come under our observation, we seek to fix the proportion of those which occur during the first year after birth, we shall find that it gives a mean of 52 per cent., a thirty-eighth part of this number being males, and a sixty-secondth part females. The proportion is thus much greater for females at this age, than for all the periods of life taken together, a circumstance readily explicable. Inguinal and umbilical herniæ are those which especially exist: to the latter the predispositions are equal, and to the former the descent of the testicles is the only additional predisposing cause, the inguinal canal being found open with equal frequency in the two sexes." (pp. 13-14.)

From the completion of the first to that of the second year, M. Malgaigne has found the proportion considerably diminished, and still more so between the ages of two and five. From five to thirteen this decrease continues in nearly an equal proportion for the two sexes; and he particularly remarks that it is between eight and nine years of age that the fewest herniæ occur. He conjectures that the explanation of this fact is to be sought in the interval which exists between the development of new exciting causes of the disease, and the cessation of those which operated as predisposing causes at the earlier period of life. From the age of thirteen to twenty the proportion is augmented in a marked manner, but involves almost exclusively the male sex; a fact which, we think with M. Malgaigne, may be rightly accounted for by the increased physical exertion of the one sex,—whether it be in the games and exercises of the better classes, or in the laborious employments of the lower —whilst the other is restricted usually from those healthful recreations of the body which are permitted at an earlier period of life, but which are prohibited as unfeminine when the ultimate development of the frame most demands them. From twenty to thirty the proportion still continues to increase, especially during the last two years, and affecting women rather than men. The rarity of umbilical and crural herniæ in females from accidental causes previous to this age (M. Malgaigne has seen but one case), together with the tendency to those forms of the disease dependent on the phenomena of puberty, and especially pregnancy, are assigned by our author as the reasons for the peculiarity alluded to. From thirty to thirty-five, no marked change takes place; but from the latter age to forty the numerical progression is nearly doubled in both sexes, and exceeds the proportion of any subsequent years. Between forty and fifty the average preponderates amongst women; but after that age men are more subject to the occurrence of rupture, the *general* proportion remaining nearly fixed up to seventy; lastly, between seventy and eighty, it drops to one half in men, and one third in women.

In speaking of the causes of rupture, Mr. Lawrence refers them to two heads, viz.: the "occasional or exciting, and those which act as predisposing causes of the complaint." These divisions should, of course, include the consideration of climate, occupation, general habits, and original conformation, to render the enquiry complete. On these subjects we propose now briefly to analyse the contents of our author's volumes.

M. Malgaigne informs us, that among the conscripts of Paris during the years 1816-23 inclusive, he remarked that of the rich one in thirty-seven was ruptured; amongst those in easy circumstances one in thirty-eight; whereas of the poor one in twenty-eight had herniæ. (p. 23.) His further consideration of the relative liability to hernia of different parts of France is more curious than important. The Celtic race, who occupy central France, are the most subject to rupture, whilst the Norman, Germanic, &c., are especially exempt. The comparative frequency of hernia in different countries is a point which involves the influence of too many casual circumstances to offer much temptation to the statistical enquirer. The employments and habits of the natives are, probably, the more correct source to which we may look for an explanation of the varieties in question; for with the enervating and relaxing influence of a hot climate, we find, more or less, amongst all classes a disposition to physical indolence. We have already noticed the relative frequency of rupture in Germany, France, Italy, and Spain, according to Juville's account; we may add that in Switzerland and Holland, the inhabitants appear especially obnoxious to the complaint; a prevalence which, as it affects the former, we think Mr. Lawrence justly coincides with Blumenbach in ascribing to the "universal practice of violent gymnastic exercises by the young lads," the more violent exercise of poising and throwing heavy weights by the same, and their practice of carrying heavy weights on their backs. As to the Dutch, we feel at a loss to account for their proneness to hernia; but we cannot but concur with Mr. Lawrence in smiling at the cause assigned by many authors (Malgaigne amongst the rest), viz., the milk, cheese, and potatoes, and especially the first, which is so common an article of diet in Holland. Mr. Lawrence may well ask jocosely how it is, "that a single Irishman should escape the united operation of the milk and potatoes?" (p. 48.)

M. Verdier found those of the "nervous and lymphatic" class the most subject to rupture. (Tab. v., p. 230.)

In turning to occupation and profession, constituting one class of predisposing or exciting causes of hernia, we do not observe that exact concordance in the different statements which we should have anticipated; we merely give the results. M. Malgaigne divides his tables into two classes: in the one he includes those employments which are prosecuted in the erect posture, and the other comprises sedentary occupations. Between the ages of fifteen and thirty-five, the proportion of the former to the latter, is as 83 to 21; between thirty-five and eighty, it is as 104 to 39 (p. 39): thus a standing employment appears to operate as an important predisposing cause to this disease. The table of M. Verdier differs in giving individual professions and trades, and is too extended to transcribe in full; we will, therefore, satisfy ourselves, by noticing a few of the leading particulars. Of 950 cases, 204 were individuals without occupation or profession, and therefore, it is to be presumed, belonging to the wealthier classes; 158 were bankers, merchants, and trades-people; 141 military, the infantry exceeding the cavalry in the proportion of three to one; a fact which, however, is doubtless accounted for by the great preponderance of the former over the latter division of the army, for it is admitted at all-hands that the exercises of the cavalry, who are called on to dispense with the use of stirrups, greatly tends to produce rupture. The other classes in M. Verdier's table include almost every variety of employment, and 129 individuals were too young to have any fixed profession or trade. (Table vi. p. 231.)

In addition to these various causes, as predisposing to hernia, we may enumerate local weakness, such as laxity of the abdominal muscular parietes and peritoneum; rapid emaciation after corpulency, (of which Mr. Lawrence mentions a remarkable case, p. 46); and the "injudicious application of tight clothing to the trunk of the body," by which the healthy action of the respiratory organs is interfered with, and the abdominal viscera are compressed and forced downwards. M. Malgaigne arrives, by a singular process of computation, at an opposite conclusion to that above cited: having remarked the altered form of the chest and abdomen produced by the tight zone worn by a Greek, he enquired of him whether rupture was common in his country, and being informed that he was unacquainted with the disease referred to, the professor conjectured that its existence is very rare in Greece, but admits that it is only a conjecture! (p. 28.) The numerical preponderance of right-side over left ruptures would appear to depend on the more ordinary employment of the right side of the body, especially when great exertion is required. This point has been, we think, satisfactorily discussed by M. J. Cloquet, in his researches on the subject. M. Malgaigne seems more disposed to attribute the peculiarity in question in part, at least, to certain circumstances noticed by Camper and Wrisberg in reference to the descent of the testicle, and occlusion of the internal ring. These anatomists found, that where only one testicle had descended, it was more frequently that on the right side than the left, and the peritoneal opening continued unclosed longer on the former side. Our author also admits that, as the result of his own observations on herniæ existing in right and left-handed persons, he found in 136 of the former class, 91 right ruptures; and in 17 of the latter, 10 left ruptures.* (p. 47.) These facts certainly favour the theory of M. Cloquet.

Before we proceed to treat of different forms of herniæ, we will pause just to see what our authors have to say respecting the general mortality in these cases. As we do not allude especially to the fatal termination of cases subjected to operation, a reference to the tables containing the proportion of the ruptured to the whole population at different periods of life, will conduct us to the attainment of the desired information. M. Malgaigne informs us that he has communicated the result of his enquiries on this head to the Academy of Sciences, considering that the subject, treated statistically, is one of the highest practical interest; in proof of which he gives us this summary of his investigation: Up to thirteen years of age, there is a numerical decrease in the proportion of ruptured individuals to *one in seventy-seven;* from which period to the age of forty the ratio gradually increases, until we find one ninth part of the population affected. So far then we may assume, that this disease exercises a very trifling, if any, influence (strangulated cases apart) upon the duration of life, fresh sufferers being added without a proportionate mortality; and this even continues to operate up to the seventy-fifth year, at which age the "ruptured form almost one third of the male population;" but, after this age, the proportion diminishes rapidly,

* These remarks of course bear especially on the inguinal form of hernia.

whence we are naturally led to infer that life, after the age above mentioned, is decidedly curtailed by the existence of hernia; a fact which should "induce us diligently to seek to perfect the palliative treatment, so as to give the ruptured part of the population an equal chance of longevity with others." (p. 21 et ante.) As M. Verdier's tables do not ascend beyond the age of seventy-eight, we are unable to compare them satisfactorily with the preceding.

We now pass on to the consideration of different forms of hernia, in which we shall continue to follow M. Malgaigne in the brief analysis we propose to give of each division of the subject, and, as we have already devoted so much space to the discussion of the statistical portions of their works, we shall confine ourselves to the selection of such points of agreement or contrast in our authors as may best merit attention.

*Inguinal Hernia.* In the introductory remarks to his lectures on this, the most frequent and important form of rupture, M. Malgaigne considers the several predisposing causes which operate in its production; some of these we have already noticed in our general remarks in the preceding pages, and we now proceed to examine the remaining. In speaking of *hereditary predisposition* to this complaint, the following are Mr. Lawrence's remarks:

"When it is stated that hernia has sometimes appeared to be hereditary, the meaning of the observation must be, that there is a certain weakness in the original formation of the parts, predisposing to the complaint, and that this defect may descend to the offspring, and, in this sense, its truth cannot be disputed. I believe that the word *hereditary*, in its application to disease, has been always used according to this interpretation; and that the employment of it, in its strict sense, has only been suggested by those who wished to show their ingenuity in refuting an absurdity of their own creation." (p. 45.)

If Mr. Lawrence mean, as we can scarcely suppose, by the above paragraph, that *no contracted* disease can be conveyed from parents to children, it is an opinion which general experience and observation contradicts; but as applicable to hernia and other local complaints of a similar nature, his remarks are doubtless correct; and we cannot conceive how any one, with the most moderate pretensions to a knowledge of physiology, could broach a doctrine so inconsistent with its laws as that deprecated by Mr. Lawrence. With this understanding, then, we give an abstract of M. Malgaigne's comments on the subject.

He observes that "it is an important question in relation to which he possesses very significant tables," and that "the hereditary predisposition is one of the most powerful influences." Of 316 individuals afflicted with hernia, eighty-seven informed M. Malgaigne that the disease existed in other branches of their families; a number which, in itself large, would probably have been augmented had the truth been known or elicited in every case. We do not consider it necessary further to individualize the degree of relationship which is given in different columns of M. Malgaigne's pages, but we may notice that by far the largest number was in a direct line from father or mother to children, and that there were many instances in which brothers were the subjects, (p. 42, et ante.) As we do not find that our other authors have any remarks on this point, we may proceed. Height and the form of the abdomen M. Malgaigne has observed to have an important influence in predisposing to inguinal hernia. To the former his attention had been first directed by an army-surgeon, and his own subsequent observations coincided with those of his informant; of seventy-eight individuals, fifteen were under (misprinted "au-dessus,") five feet (French ;) twenty-one between five feet, and five feet two inches, and forty-two above the last height. This yields a proportion very unfavorable to height; a fact which M. Malgaigne connects with "a proportionably greater feebleness of constitution." We should guess our author was not a very tall man. With respect to the development of the abdomen, he has found that the flat-bellied comprise more than half the ruptured, whilst the moderately fat and very aldermanic are comparatively exempt; for the definition of a fourth form of abdomen, entitled "triple saillie," we must refer our readers to the original, and to the City of London.

The immediate or "determining" causes of inguinal hernia, is a subject of sufficient importance to demand some attention. In a great many cases it is of course impracticable to trace the exciting cause of rupture; indeed in many instances none can be said to have existed; but of those which can be determined, the class which contains the highest numbers in the tables of both Verdier and Malgaigne, is that which comprises those who have suffered from lifting heavy weights. Falls, coughs, asthma, &c. are fruitful causes of this, as indeed of other forms of hernia; but next in numerical succession to the first-mentioned cause in M. Verdier's table, is habitual constipation and hemorrhoids. The truth of this last observation our own experience leads us to corroborate; and we should think most practical surgeons must regard constipation as one of the most common causes of what is termed spontaneous inguinal hernia. The exertion constantly employed by those who are habitually constipated to relieve the bowels of their contents, produces a decided tendency to enlargement of the inguinal rings and canal, which is most favorable to the descent of a hernia; and this as frequently in women as in men. We are therefore rather surprised to find that M. Malgaigne overlooks this in his enumeration of the determining causes of the form of rupture in question.

In his next lecture M. Malgaigne comprises the different aspects under which inguinal hernia makes its appearance: he adopts the ordinary division into that form which takes place previous to the closure of the opening of communication between the general sac of the peritoneum and vaginal tunic of the testicle (congenital), and that which takes place after this process is completed. The former of these he subdivides into herniæ which are inclosed in the serous covering of the testicle, and those which do not descend below that portion of the vaginal tunic which invests the spermatic cord. To these heads may be added the encysted hernia of Sir A. Cooper, amongst those forms of rupture proper to infancy. This arrangement tends to a misapprehension which is not intended by the author; for it has long since been shown that the impression of Mr. Hey (who first described this form of hernia,) was incorrect, when he represented it as peculiar to the earliest period of life; the fact being that though the vaginal tunic forms an external sac, the internal or proper sac of the hernia is derived, as in other instances, from the peritoneum at the closed internal ring. M. Malgaigne justly condemns the term "congenital" as it is usually employed, for it implies that which in truth is scarcely known to exist; but in his speculations upon the question of why a hernia should not descend prior to birth as well as the testicle, he seems to have forgotten that the latter is subjected to a peculiar force dependent on the gradual contraction of the gubernaculum. He well may add that the violent efforts of crying, coughing, &c., (the common cause of hernia in children,) cannot operate before the lungs have been inflated by the first inspiration. The influence of long-continued pressure on the testicle, in producing wasting, or perhaps rather arresting development, has been frequently remarked by M. Malgaigne in testiculo-vaginal rupture. Mr. Lawrence's remarks on the employment and proper application of trusses in these cases demand the attention of the young surgeon; we find that they are closely followed by the French professor.

"Before the surgeon applies a truss for an inguinal or scrotal rupture in a young person, he must satisfy himself not only that the protruded parts are fairly replaced, but that the testicle has reached its normal situation in the scrotum. A rupture may take place in an infant when this gland has not yet quitted the abdomen. . . . . The application of a truss to a young subject, thus circumstanced, might prove injurious by retarding the descent of the testicle. If it should have arrived only so far as the groin, the pressure of the pad on the gland may be attended with still worse effects." (Lawrence, p. 573.)

We pass over the general remarks of M. Malgaigne on inguinal hernia as containing nothing calling for notice, until we arrive at the disputed question regarding the seat of stricture in the strangulated forms of this rupture. We had looked forward to this when we first opened his book, for in the preface we found it announced that "a new doctrine" was to be broached on the subject. This new doctrine proves to be that the true seat of stricture is the neck of the sac! We leave our readers to judge of the *novelty* of this explanation, whilst we proceed to give a few of the professor's remarks. We have said that a variety of opinion has existed, and, we may add, still exists upon this point; for example, our author admits that Dupuytren thought the neck of the sac was the most frequent seat of stricture, whilst Velpeau considers that when such is the case, it is an exception to the rule; and Sir A. Cooper attributes much to the constricting power of the rings. M. Malgaigne justly remarks that in operating it is comparatively of such trifling importance to decide on the tissue which is producing strangulation, provided the surgeon is able to release the contents of the hernial tumour, that it is rarely a subject of investigation (nor could it be very easily) at that time; and if a fatal termination of the case permit an autopsy, the division of textures is too general to allow of a ready determination of that which had been the cause of the mischief. What is also said by our author respecting the condition of the testicle is certainly reasonable, namely, that were the seat of stricture at the rings, this organ ought to suffer by compression of its vessels, &c., which traverse the inguinal canal. These and other considerations, deduced from dissection of old herniæ, have led M. Malgaigne to believe that the neck of the sac, altogether independent of either ring, is the seat of stricture: but the subject is of sufficient interest for us to examine severally the opinions of the different authors whose words we have before us. First of M. Verdier. In his chapter on strangulation we find the following remarks, which are all we have been able to glean from his copious volume, on the subject.

"At all times when called to the examination of a strangulated hernia, the first care should be to form a clear idea of the precise seat of the constriction to which the viscera are subjected; more especially as it may exist in all the parts whereby the organs which form the tumour are inclosed. In recent herniæ which have taken place spontaneously, it depends almost always on the attraction of the aponeurotic fibres which embrace the viscera. But if the hernia is an old one, the fibrous bands are too feeble to produce such an effect. It is then on the thickness and firmness (solidité) of the neck of the sac, that the compression of the protruded organs depends. Sometimes the aponeurotic opening and the neck of the sac may concur in constricting the viscera." (p. 161.)

We have made a literal translation of the above passage, in order to avoid misinterpretation of our author's meaning, which is a little indefinite in consequence of our not being able to fix a determinate signification or position to the aponeurotic fibres he mentions. If by "aponeurotic *opening*" he intends us to understand the funnel-shaped prolongation of the fascia transversalis, usually denominated "internal *ring*," we think he is right; at any rate he is not peculiar in the opinion that this is not an infrequent seat of stricture. But let us turn to the observations of M. Vidal, who treats the subject of hernia generally in a concise and practical manner.

"Strangulation," he says, "is often produced by the ring, [external ?] but even more frequently perhaps by the superior orifice of the canal, [internal ring ?] or by the neck of the sac. Congenital hernia is more often strangulated by the neck of the tunica vaginalis. The superior orifice of the canal (inguinal) being formed on its inner side by the internal portion of the transversalis fascia, mingled with the tendinous insertion and inferior fibres of the internal oblique and transversalis muscles, it may be conceived that the contraction of these muscles may contribute in augmenting the constriction. The inguinal is the only form of hernia which presents this disposition of stricture, and in which a spasmodic strangulation is rendered probable. This hernia is, doubtless, that one most frequently meets with sacs having several necks, and in consequence, numerous points of strangulation." (p. 29.)

The observations of Mr. Lawrence may be embodied in the following brief quotation:

"In performing the operation for strangulated hernia, and especially in liberating the protruding parts from pressure, which causes strangulation, we must bear in mind that the ring of the obliquus externus is not the only, perhaps not even the most frequent seat of the stricture; that the parts may be confined at the upper aperture of the inguinal canal or by the neck of the sac; and that, occasionally the stricture is below the canal, and altogether independent of it." (p. 265.)

Further on, in proof of his impression that the external ring is sometimes the seat of strangulation, the same author gives us a valuable hint as to the diagnostic marks by which we may determine this important point: he says,

"When the stricture is formed by the ring of the external oblique, the swelling of the rupture does not extend beyond that point. The inguinal canal is soft, compressible and indolent; while the ring is distended, tight, and hard. When, on the contrary, the strangulation is at the neck of the hernial sac, the inguinal canal is full, hard, and painful, presenting a firm cylindrical tumour extending from below obliquely upwards and outwards." (p. 266.)

So much for this disputed point: we have only to add our belief that, though the neck of the sac is probably the most frequent seat of stricture in oblique inguinal hernia, both the external ring, and conjoined tendinous insertion of the internal oblique and transversalis muscles are occasionally found to strangulate the protruded intestine or omentum. Indeed, we can scarcely otherwise account for the frequent examples that we have of the favorable result of the taxis, when aided by measures the direct tendency of which is to paralyse muscular contraction, such as bloodletting, emetics, hot bathing, and even the tobacco enema.

The next subject discussed by M. Malgaigne, is "the conditions of the radical cure of inguinal ruptures." He considers that the desirable result of a permanent cure, is only to be hoped for in the oblique forms of inguinal hernia; and that even in these there are several important distinctions which it is essential clearly to define and establish. In infancy, for example, he considers that a radical cure is most easily obtained, as the indication is only to complete that which nature has left unfinished, namely, obliteration of the communication between the general peritoneal cavity and the tunica vaginalis. The natural tendency to this effect at a very early age, doubtless materially aids the efforts of the mechanist or surgeon; but great care should be taken that the adaptation of the means employed do not aggravate instead of cure the existing mischief, as Mr. Lawrence has pointed out may be the case. M. Malgaigne has found great success attend the proper application of mechanical means between the ages of thirteen and thirty-five; a fact which he attributes to the frequent occurrence of hernia from bodily exertion at this period of life. This explanation is, no doubt, just; inasmuch as the purely accidental production of rupture indicates less of a predisposition to the complaint, than where the disease is spontaneous, which is the case later in life. But some little credit must also be assigned to the renovating power of the constitution, which is more peculiarly the attribute of youth.

We pass by the '*history*' of trusses, to see if we can discover any thing practical worth extracting from our authors on the subject of those now employed. After speaking of the soft leather and linen trusses, which are now rarely seen, M. Malgaigne discusses the merits of the French and English contrivances in steel; and the preference he gives to Salmon's simple and ingenious piece of mechanism, must be gratifying to the inventor, as it is candid in the professor. We say this because he confesses having commenced his examination and practical testing of their relative merits, with a decided predilection for the French contrivances, and a peculiar antipathy to Salmon's truss, based upon a supposition that its theory was not consistent with the anatomical arrangement of the parts to which it was applied. He appears to have tried in vain to modify Salmon's apparatus so as to make it meet his own preconceived notions on the subject; and at last candidly confesses that, with the exception of the badness of the steel, (the last exception we should have anticipated from a continental critic,) it is the most perfect contrivance that has yet been invented. We do not observe that the long and complex account of what a truss should be according to M. Verdier, contains anything particularly worthy of notice. Amongst the various forms of elastic and inelastic pads described by M. Malgaigne, he notices those furnished with spiral springs, and others made with caoutchouc and distended with air; the former he has found only applicable in umbilical ruptures; and the latter, though excellent when new, sooner or later fails from the gradual flaccidity consequent on the invariable escape of the air; he has found them applicable to some cases, but does not specify what sort. After denouncing the compresses of solid wood or ivory for inguinal hernia, M. Malgaigne informs us that he has procured some pads of solid caoutchouc which are in every respect desirable, and appear to be durable: at the same time he admits that a nucleus of wood, well and evenly padded with wool, and covered with soft leather, answers the purposes for which it is destined nearly equally well. We think these points are of great importance in the cure of hernia as well as for the comfort of the patient; and should receive more of the attention of the surgeon than they always do. Mr. Lawrence (who does not appear to give a preference to any particular form of truss, provided it fulfil certain desirable objects which he points out,) considers that the size and form of the pad require especial consideration in reference to the case to be treated; and he justly indicates the evil consequences of neglecting to adapt a compress of a proper degree of convexity; a too convex pad not only often leads to mischief by permitting the escape of some portion of the usual contents of the sac, but also "by pressing the external part into the opening, it keeps them distended, and prevents that contraction on which a radical cure depends." (p. 96.)

The space and attention which have been devoted by M. Malgaigne to the farther consideration of the *form* of hernial pads is an indication of the important share he assigns to them in the radical cure of rupture; we should therefore scarcely do our author justice if we did not analyse his views; the more especially as his experience seems to have been extended, and as he appears to have had but one object in view, that of testing the practical application of such contrivances as theory suggested. First, in speaking of cases of indirect hernia, traversing the whole inguinal canal without having much distended it, and descending into the scrotum, he remarks that "it is upon the entire canal that the pressure should be made, and especially towards the abdominal orifice (internal ring)." (p. 117.) This is satisfactorily effected by the last phalanx of the thumb; but then, as the slightest movement in either direction would suffice to permit the escape of the hernia, M. Malgaigne recommends that the pad should be half as long again as the canal, and half as wide again as the thumb: this surface he has found sufficiently extended for ordinary cases. There should likewise be convexity enough to preserve the walls of the canal in contact. Again, in dealing with an indirect hernia, in which the canal is much dilated, allowing of the ready escape of the bowel from the laxity of the walls of the canal, the small convex pads just described are useless, and obnoxious to the objection we have already quoted as noticed by Mr. Lawrence. In these cases M. Malgaigne employs pads of at least double the breadth of the thumb, "and having an ovoid convexity which is bevelled off toward the internal ring." (p. 119.) But, thirdly, a hernia may traverse the whole canal without causing much dilatation; yet, if it occur in an old man, the tissues may be rendered lax and weakened by age, and even more by obesity: in such cases our author thought that, as he had seen ruptures disappear in individuals nearly seventy years of age, that the small convex pads already noticed might be as successfully employed as in the young. Experience, however, taught him that, on the contrary, these pads first produced absorption of the subcutaneous cellular tissue, and subsequently pressed too forcibly on the weakened aponeurosis, so as ultimately to produce farther distension in place of obliterating the ring. In such cases, therefore, he has found it requisite to employ, for the purpose of procuring a radical cure, very large pads, *i. e.* of three fingers' breadth, and four fingers' length, with a very moderate and uniform convexity. (p. 120.) Some cases, says M. Malgaigne, may, however, be met with which call for a peculiar treatment; "those, e. g., which have become almost or altogether direct:" then the ordinary pressure is of no avail; but a pad framed so as to press in the integument, in the same way as would be effected by one or more fingers, (the prominence in short being digitiform,) has been found efficacious, but they are only to be regarded as "an extreme means when all others have failed." With regard to the *form* of the pads adapted to different cases, M. Malgaigne acknowledges that he has been forced to renounce the circular, semicircular, and elliptic as useless; but that he has found a hernia retained by one of a very singular shape, after a fair trial had proved the inutility of the above. It is described as "*une pelote à bec de corbin*," (p. 122,) i. e. a triangular pad, of which the inferior and most acute angle "rests on almost the whole upper border (hauteur) of the pubis." He does not attempt to explain satisfactorily the modus operandi of this pad; but he says, "I am willing to be satisfied with facts, so that I repeat, you will not succeed in retaining very difficult ruptures with any but the beak-shaped pad, which should, moreover, present a very extended surface." (p. 123.) From a careful consideration of the form of the inguinal canal, in connexion with the influence of pressure, M. Malgaigne has been led to employ three different kinds or degrees of convexity in the construction of pads; of the first the form is elliptic, and the convexity uniform in every direction, by which means an uniformity of pressure is exercised over the whole extent of the canal; this pad is peculiarly applicable where the subject of the complaint is young, the aponeurotic tissues not very strong, and the muscular efforts moderate; a slight increase of convexity towards the centre produces a modification by which the pressure is more decided upon the middle of the canal. Secondly, an oval pad, the greatest convexity of which corresponds to the internal ring: the effect of this arrangement is obvious; and it is found best adapted for individuals in whom the tendinous aponeuroses are strong and resisting, and the muscular efforts considerable. Thirdly, the same as the preceding, but with the addition of the greater transverse convexity of the pad being approximated to the inferior border of the canal: the end proposed to be fulfilled by this form is that, in persons whose muscular development and violent efforts permit the parallel fibres of the transversalis and internal oblique muscles to separate the walls of the inguinal canal, and elevate the pad, sufficient pressure may be made at the inferior border of the former, so as to counteract the above-mentioned tendency, (p. 125;) in short, to concentrate the principal operation of the support to the interval between the lower border of the conjoined tendons and Poupart's ligament; an indication which we should think the contrivance in question well adapted to answer.

In the two succeeding lectures M. Malgaigne treats of the union of the pad and spring of the truss, discussing the relative merits of the fixed and moveable pads; and also notices the accessory appendages necessary to the securing of trusses: but we must pass over these subjects, and will briefly recapitulate, after our author, the directions which he has given for the selection of the appropriate means of support adapted to various forms of rupture.

"1. For a simple, inguinal hernia the best spring is the English spring, which embraces the body on the sound side. 2. If the hernia is oblique and easily retained, the moveable pad should be selected; but where the reverse is the case a fixed pad is preferable. 3. There should be always a decided interval between the extremity of the spring and the internal surface of the pad, whether it be procured by an intermediate pad, or only by augmenting the thickness of the compress itself. 4. It is on the form of the compress that the retention of a rupture principally depends. For oblique herniæ the pad should cover the whole canal, making pressure, according to circumstances, either directly upon the centre of the canal, or with an inclination towards the internal or external ring. 5. In direct herniæ the preference should be given to the triangular pad, (à bec de corbin.) 6. In very thin subjects, the spine of whose pubes is very prominent, and unable to sustain the pressure of the ordinary pad, that filled with air is a resource. 7. The leather strap and that which passes beneath the thigh should never be too tight. The rupture ought to be retained by the power of the spring, of which the other parts of the truss are only auxiliaries. 8. Every truss that allows the escape of the hernia does more harm than good. 9. If a truss hurt or incommode the patient it is bad; at the same time, it must be admitted that there are some herniæ which are extremely difficult to retain, and which therefore form an exception to the above rule, great constraint being the unavoidable result of the application of the necessary apparatus." (pp. 155-6.)

Lastly, before quitting the subject of trusses, we may notice the result of M. Verdier's experience with respect to the radical cures effected by their use at different ages. He thinks that, prior to the age of puberty, we may count upon a permanent cure in three fourths of the cases. During the early adult periods, provided the rupture be recent and the result of accident, about two thirds are relieved. As age advances the proportion decreases; so that, of subjects above forty years, not more than one in ten is radically cured. (pp. 150-1.)

Inguinal hernia in females is next discussed by M. Malgaigne. Nearly the whole lecture is occupied with the subject of the relative frequency of this form of the complaint in men and women; and we are not a little startled by the announcement, that it is more common in the latter than the former; i. e. that the relative proportion of inguinal to crural ruptures is greater in women than in men. This, we need scarcely observe, is so completely at variance with all our preconceived notions, and with, we believe, the experience of every accredited writer on the subject, that we felt not a little curious to see how M. Malgaigne disposed of the mass of evidence which he was certainly bound to face and undermine, if not overthrow, before he could expect credit for his own assertions and inferences: we will accompany him through his lecture for this purpose. After noticing the general opinion respecting the predominance of crural hernia in women, he addresses himself to an analysis of the testimony on which it is founded, viz. that of surgeons and truss-makers. First, with respect to operating surgeons: their evidence is, that almost all the cases of strangulated hernia which occur in women are of the crural class. This fact is undeniable, and therefore undenied: "but what," says M. Malgaigne, "are we to infer from this? simply that crural ruptures are more liable to strangulation than others." He of course denies the propriety of including strangulated and simple herniæ in the same category, and with at least some show of justice. But then truss makers, who deal with reducible ruptures, also agree that those of the inguinal class are very rare in women, the largest proportionate calculation opposed to his theory being that of the London Truss Society. Of six hundred and ninety-three females with inguinal and crural herniæ, there were only forty-four belonging to the latter class—a proportion of one in fifteen. Now, if M. Malgaigne's assumption be correct, viz. that the compilation of these important tables was left exclusively to the judgment and opinion of the instrument makers exercised upon the individual cases, we perhaps might not be much disposed to quarrel with his inference regarding their value and credibility; but as we have reason to believe that such is not the case, we can hardly admit his summary method of dismissing this evidence by denying the capacity of those upon whose authority the statistical table was drawn up. But again, especial attention has been devoted to the subject by a M. Nivet, whom our author styles "a very distinguished *interne* of the Parisian hospitals;" and his calculation is still in favour of the prevalence of crural hernia, in the proportion of sixty-seven to forty. All that M. Malgaigne says to this evidence is, that it is more in his favour than the generally-received opinion, and hints that the calculation was undertaken to verify the old notion, and also that the period of life at which the cases were examined was more favorable to the existence of crural ruptures. But his toughest opponent he acknowledges to be M. J. Cloquet, whose calculation was made from examination of the dead body: of 121 crural and inguinal herniæ in women, there were but 42 of the latter class. It would indeed appear impossible to get over this obstacle to the establishment of his own doctrine; but M. Malgaigne, nothing daunted, denies the correctness of M. Cloquet's calculation, on the ground that he included every crural hernia, however small, and whether only commencing or obliterated! We may lastly remark, that the combined authority of all our other authors is opposed to M. Malgaigne. M. Verdier says, "inguinal hernia, although *very rare* in women," &c. (p. 28.) Vidal admits that the proportion of inguinal herniæ in females would probably be augmented if the diagnosis were always correct; for in cases of enlarged and relaxed external ring, the hernial sac may be directed over Poupart's ligament, so as to occupy the position of a femoral hernia, instead of the more ordinary course into the labium pudendi. (pp. 12-13.) Mr. Lawrence refers to the tables already noticed, as well as to those of M. Monnikoff, who found, in 885 individuals affected with inguinal hernia, that only 175 were females. (p. 229.) Before we conclude our remarks on this subject we must, in justice to M. Malgaigne, give the result of his own (as we have no reason to doubt) impartial examination of the subject. His first observations were made in 1835, when, much to his own surprise, he found that 54 out of 62 females (with the two forms of rupture in question) had inguinal hernia! This proportion, he acknowledges, exceeds any that his more recent observations have given him; but still he is borne out in his conclusion, that "inguinal is more frequent than crural hernia in women." (p. 178 et ante.)

With regard to the predisposing and determining causes of inguinal rupture in females, the same author remarks, that the comparative narrowness of the peritoneal prolongation in the inguinal canal, (known as the canal of Nuck,) renders females less liable to the complaint than males—the proportion being about one fourth. This is a little augmented in the first two or three years, subsequent to which period, until about the twentieth, the fresh cases are rare, though the crural form is even more so; and even after this period, when the important influence of childbearing is taken into account, and the gross number of herniæ greatly increased, still the inguinal are predominant. As a disposing cause to rupture generally, M. Malgaigne considers that pregnancy absorbs all those circumstances and special influences which we have already noticed in connexion with this disease in males. The relative number of accidental and spontaneous inguinal herniæ he has found about the same in both sexes. One remark which our author makes in reference to trusses for females is worth extracting: where the spinous process of the pubes is very prominent, a special depression on the pad for its reception will be found very efficacious in aiding its proper adaptation:

We pass by the "interstitial or incomplete inguinal hernia," (i. e. where the sac remains in the inguinal canal,) which is more particularly treated of by Mr. Lawrence (p. 220) and M. Vidal, (p. 13,) the authority of M. Goyrand being quoted as having given the fullest account of it in the fifth of the *Mémoires de l'Académie de Médecine*, 1836; but the crural form of rupture will require a short notice at our hands.*

A lecture is devoted by M. Malgaigne to the consideration of the diagnostic marks by which crural and inguinal hernia may be distinguished. He first of all quarrels with all the methods hitherto recommended, before he proceeds to establish his own more perfect system; and first of Sir A. Cooper's signs, viz. 1, The position of the sac's neck above the pubic spine in inguinal hernia, and below and external to it where the rupture is crural; 2, The position of the crural arch in relation to the neck of the sac. With respect to the latter, our author considers it only applicable where the parts are exposed by operation; and as to the former, he thinks that, from the dragging of an inguinal hernia on the inferior pillar of the ring, the neck of the sac is frequently found *beneath* the spine of the pubes; and he denies that it is usually internal to the same point, (an assumption, by the way, of his own, that Sir A. Cooper has stated the affirmative;) and further makes the captious and foolish remark, that incomplete inguinal herniæ seem to have escaped Sir Astley's notice. It would not require a conjuror to detect that these were not crural: but, to proceed. The diagnostic signs of M. Amussat he thinks nearly as fallacious. This surgeon recommends that a line be drawn from the anterior superior spine of the ilium to the pubic spine, and that all herniæ above this line be set down as inguinal, and all below it as crural herniæ. We need not stop to show the fallacious nature of this

* The characters of this form of hernia have been described and delineated by Sir A. Cooper in the first part of his work: a fact which Mr. Lawrence remarks might have saved MM. Goyrand and Dance their reclamations on the subject of priority of discovery in 1835.

test. M. Nivet, prior to his researches already noticed, established a modification of the above, viz. that of tracing Poupart's ligament from the pubic to the iliac spine, and then classing the ruptures according to their relative position: in truth, says M. Malgaigne, this is a sufficient test, *if* you can trace the ligament in question. But it is time that we examine the plan of proceeding which is to obviate all these difficulties.

"Suppose then," says M. Malgaigne, "a hernia of the most equivocal character, a tumour in the midst of the groin, but a little approximated to the pubes, projected on coughing, returnable by pressure, but so suddenly as to defy detection of the point of disappearance," &c., &c. [In short, suppose a case which none of the tests already enumerated would suffice to determine, then the following is the rule of proceeding:]

"You return the rupture; you discover with the right index finger the pulsations of the femoral artery; and, applying the extremity of the finger to the inner side of this artery, you must press backwards until you feel the pubes. Sometimes, in thin persons, you may feel at the same time the crural ring open, with its boundaries of Poupart's ligament in front, the os pubis behind, and femoral artery externally,* then you need proceed no further; in the normal condition the finger can never penetrate thus into the crural ring. But suppose you are dealing with a fat subject and a very small hernia, and that the ring is too deep and narrow to admit the finger, you must press on the pubes, feeling the pulsating artery on the outer side, and then direct the patient to cough. If the impulse is felt against the finger, and the rupture, nevertheless, does not descend, you may conclude that it is crural hernia; but if there is no such impulse, the hernia, on the contrary, making its exit above, you may be satisfied of its inguinal character. But, again, the exit and impulse spoken of may take place together; then, whilst the right index finger is closing the crural ring, place the left thumb transversely about three lines above it, and direct the patient to cough: if the rupture be inguinal, it will be retained; but if crural, it will descend so as to leave no doubt of its character." (p. 188 et seq.)

The other difficulties which present themselves are met, and are said to be obviated by corresponding instructions, which our space will not permit us to transcribe. We leave our readers to judge of the value of these directions, which can only be applicable where the hernia is reducible; where the reverse is the case, our author admits that the surgeon must be guided by tracing the sac to its point of exit.

Of the predisposing causes to this form of hernia, M. Malgaigne reckons frequent pregnancy amongst the most influential, (p. 194:) and he states the results of his experience to be opposed to that of Sir A. Cooper; M. Malgaigne having found that the greater proportion of the patients whom he has examined, attribute their disease to a sudden effort or a blow received on the abdomen. (p. 196.) It is in such cases that he has found the stricture formed by the ruptured cribriform fascia; indeed, he denies, in this as well as inguinal hernia, that the rings have anything to do with the strangulation. (p. 198.) We are, by no means, disposed to pin our faith on M. Malgaigne's experience on this point. He thinks the ordinary method of distinguishing between inguinal hernia and varicocele insufficient to distinguish between the latter disease and crural hernia; he therefore directs that, instead of returning the rupture, pressure should be made upon it, so as to fix it against the pubis, and then that the tumour should be pressed with the thumb of the

* We presume M. Malgaigne has not forgotten that the *vein* forms the external wall of the ring?

other hand. Under this manipulation the varicocele will disappear, the blood flowing into the inferior veins, whilst the reverse would be the case with a hernia. (p. 204.) We find, on reference to M. Verdier's tables, that he gives, as the proportion of cases of crural hernia which have fallen under his notice, one hundred in females, and fourteen in males. (p. 240-1.) As to the palliative and radical cure of this form of rupture, M. Malgaigne denies the possibility of the former, and admits the difficulty of applying any form of truss which shall be fully efficacious even in retaining the protruded intestine; it should be as accurately adapted as possible to the form of the groin, and sufficiently prominent to make pressure directly upon the crural ring.

M. Malgaigne does not seem able, from his own experience, to throw much light on the statistics of umbilical hernia. It would appear that the tables, which have been compiled on the most extensive scale, do not agree with the results obtained by Sir A. Cooper, who found this form of rupture the most common, inguinal excepted. Such, likewise, appears to be the conclusion to be drawn from M. Verdier's tables, who gives as the relative proportions of crural and umbilical hernia 156 of the latter and 114 of the former; 97 of the umbilical ruptures occurring in women, and 59 in men. M. Malgaigne was at first inclined to this opinion also, but subsequent experience has disposed him to coincide with the tables of Monnikoff and others. In regard to the age at which this form of hernia occurs, M. Malgaigne's results are curious and interesting. Whilst in the mass these ruptures are more common in women than men, he has found that the proportion is reversed according to the period of life: thus, from birth to the age of thirty, there are twice as many cases in males as in females, the converse holding good from the latter age to eighty: the explanation given of this apparent anomaly, is that the newly-born of the male sex are "enormously more subject to the complaint than females; he believes they are sometimes strictly congenital." (p. 223 et ante.) The fallacious impression which is occasionally received regarding the exact position of an umbilical rupture when returned, M. Malgaigne attributes to the firm cicatrix resulting from the union of the obliterated vessels and urachus. Speaking of the cure of these cases, he recommends the employment of a compress which must be accurately fitted *into* the umbilical depression so as effectually to *cork* it up: the size of this plug must be gradually reduced as the annular opening diminishes, and it should be confined by adhesive plaster carried twice and a half round the body. (p. 232.) A ball of caoutchouc he has found to answer the purpose very well. Lastly, of ruptures escaping by the linea alba, M. Malgaigne has never seen them uncomplicated with other herniæ, and he believes it to be rarely the case that they exist alone. The largest that had occurred in his practice, was equal in size of a man's head. The radical cure of them he considers hopeless. In the other and more rare forms of rupture, such as lumbar, obturator, &c., our author has had no experience.

In the preceding analysis, we have unavoidably omitted much relating to the subject of hernia, although we have followed M. Malgaigne pretty closely through the pages which compose his lectures; but it would be idle to attempt to touch upon everything within the space allotted to our task; we shall, therefore, confine our closing remarks to a few points of interest connected with the treatment of strangulated hernia generally. Mr. Lawrence's observations on the various means recommended and employed to aid the taxis in these cases, are so excellent, so essentially practical, that we turn to them with confidence that we shall have offered to us nothing but the sound advice of an able and experienced observer. With respect to *purgatives*, he remarks, "that experience has taught him to repose very little confidence in them: they are not only inefficacious, but actually prejudicial, in the inflammatory strangulation; but in large and old herniæ, where an accumulation of fecal matter, from torpor of the intestine, is the cause of strangulation, and the symptoms are of the chronic kind, purgatives may be employed with success, and those of an active description," &c. Again "an omental hernia is another exception to the general doctrine on the subject of purgatives: if we can clear the intestines completely, the operation will seldom be necessary." (p. 160 et ante.) In speaking of that powerful agent, the *tobacco enema*, Mr. Lawrence quotes the contradictory opinions and results of the practice of different surgeons: thus, M. Velpeau, after having been once successful, tried it in twenty-five cases without advantage; whilst Mr. Key thinks that the objections raised against it are owing to a want of a due precaution in proportioning the dose to the power of the patient. Mr. Lawrence himself says, "we cannot speak so favorably of the safety, as of the power of this remedy, which has destroyed life in some instances, and probably been injurious in others....Venesection is preferable to it in the early period of strangulation, while the great depression of the vital powers makes us hesitate to use it in a more advanced state of the malady." (pp. 164-5.) Of the dashing cold water on the patient, Mr. Lawrence thinks but lightly; but he ranks the topical application of cold as inferior in efficacy only to venesection and the tobacco, in the treatment of strangulated hernia.

As M. Verdier is a strong advocate for the use of cold effusion in the cure of hernia, we should not do him justice without noticing his observations on the subject. Assuming the production of hernia to be dependent for the most part on the relaxed condition of the muscular and tendinous structures, forming the abdominal parietes, he considers the *douche* bath as peculiarly applicable in restoring their wonted tone. His attention having been called to the efficacy of this remedy by a case of strangulated hernia which was thus reduced under the care of Petit, he employed it without effect for some time in recent cases of reducible hernia; but further experience induced him, after neglecting it for a long period, again to have recourse to the douche, which he now administers in the following way:—"Experience," he says, "has taught me that the treatment of ruptures by cold affusion should be continued for at least twenty days, during which, the patient should each day, and without interruption, receive the douche in the morning." Further on he adds, that the column of water should be directed on the part affected for sixteen or eighteen minutes continuously. If the hernia be scrotal, it should be thrown upon the inside and middle of the thigh of the same side, in such manner that the scrotum and groin may get the benefit of the reflected jet which would thus fall in a sheet over these parts. When the inguinal canal and rings have recovered sufficient tone to retain the hernia, the douche may be applied to the abdomen generally. The directions for the treatment of other forms of rupture are similar; but it is impressed on us that the column of water should not be thrown point-blank on the affected part, but at an inclination of about forty-five degrees. The beneficial effects of this treatment are illustrated in the eleventh section of the third chapter of his work, in which several cases of different forms of hernia are described as radically cured by it. (p. 213, et ante.)

In the after-treatment of strangulated hernia, which has been subjected to operation, Mr. Lawrence justly dwells on the great importance of procuring a free evacuation of the bowels by gentle purgatives; and remarking upon the persistence of inflammation when once established, even though the exciting cause be removed, he adds that we should be prepared with the necessary antiphlogistics to control this common cause of the fatality of these cases. It is these observations which lead us to notice a form of treatment which we have seen attended with most satisfactory results, viz., the exhibition of small doses of calomel and opium, combined as occasion may require with gentle laxatives or enemata, and repeated every one, two, or three hours, according to circumstances. We do not mean to advocate this treatment to the exclusion of bloodletting, local or general; but we feel confident that the beneficial control exercised over the circulating system by the above combination tends greatly to arrest or even prevent the onset of peritonitis, as well as to promote a healthy action in the intestines, and to subdue general nervous and vascular excitement.

In concluding our notice of the volumes before us, we ought perhaps to apologise to our readers for the length of the article in proportion, (to retain our statistical phraseology,) to the paucity of new facts; a deficit which has also struck us, considering the pretensions of our French brethren. But in truth, former investigators (and we justly rank Sir A. Cooper amongst the first,) have left but little for us to add in the anatomy, and we may almost add surgery, of this branch of practice. With respect to the relative merits of our authors we may remark that, with a large share of vanity even for a Frenchman, and no lack of self-sufficiency in the execution of his task, M. Verdier gives us in his volume much that is useful, though in a vastly more bulky form than was essential. M. Malgaigne has handled his subject as if he intended and expected to have thrown a new light on almost all he touched; but, whatever may have been the impression on his numerous audience, both young and old, students and practitioners, we cannot say that we have been startled by any very remarkable discoveries; but, on the contrary, it has continually occurred to us in collating his works with Mr. Lawrence's to observe the remarkable coincidence in opinions, anthorities selected, and practice recommended, between the two authors. We trust we do not make this observation either invidiously or uncharitably; if the authority had been mentioned we should have esteemed the sound judgment of M. Malgaigne in following our countryman; but not having once met with Mr. Lawrence's name in the lectures of the French professor, we cannot help concluding that he is either culpably ignorant of our medical literature, or that the coincidence in question is very singular. Of Mr. Lawrence's copious, systematic, and comprehensive work, we can only reiterate that it is all the student and practitioner can desire.

